# Airborne Survival of *Escherichia coli* under Different Culture Conditions in Synthetic Wastewater

**DOI:** 10.3390/ijerph16234745

**Published:** 2019-11-27

**Authors:** Wing Lam Chan, Wing Tung Chung, Tsz Wai Ng

**Affiliations:** Department of Biology, Hong Kong Baptist University, Kowloon Tong, Hong Kong; winglam1112@gmail.com (W.L.C.); 14221845@life.hkbu.edu.hk (W.T.C.)

**Keywords:** airborne survival, bioaerosol, wastewater, culture conditions, membrane fluidity, fatty acid composition

## Abstract

Bioaerosol generated in wastewater treatment plants has potential to harm human health. Survival of bacteria in bioaerosol during suspension is one of the major factors that affect its biological risk. It is hypothesized that bacteria grown in different wastewater have different physiology and lead to variation in airborne survival. This study investigated the relationship between the cultured conditions and the bioaerosol survival. Synthetic wastewater was used as the culture medium to simulate the water quality of wastewater. *Escherichia coli* BW25113 were cultured in different conditions, including growth salinity, growth temperature, growth pH, and presence of pesticide. The fatty acid composition and the reduction in airborne survival of the *E. coli* cultured under these conditions were determined and compared. Results showed that increasing growth salinity and temperature led to a lower reduction in airborne survival of *E. coli.* *E. coli* cultured at pH 6 had a higher reduction in airborne survival than those cultured at pH 7 and 8. Moreover, a correlation was observed between the membrane fluidity (fluidity index) and the reduction airborne survival for both aerosolization and airborne suspension. A link between culture conditions, bacterial membrane fluidity, and airborne survival was established. Culture conditions (wastewater quality) that lead to a low membrane fluidity of bacteria increase the airborne survival of bioaerosol, and vice versa. This provides a new aspect to evaluate bioaerosol survival and improve assessment on biological risk of bioaerosols.

## 1. Introduction

Wastewater treatment plants (WWTPs) are a major anthropogenic source of bioaerosol. Different processes in WWTPs, such as bubble bursting, scum, and foam formation lead to bioaerosol generation [1,2]. The generated bioaerosol can be dispersed by the downwind movement [3,4], which causes potential hazards to human health, especially infectious disease, respiratory diseases, and cancer [5,6,7,8,9,10]. For example, there are several bioaerosol transmitted infections, such as influenza, winter stomach, and tuberculosis [5,10]. Moreover, airway inflammation is also caused by inhalation of specific toxins, pro-inflammatory agents, or allergens [5]. Oncogenic viruses and other biological agents such as mold can increase the risk for certain cancers [5,8,9]. Iven et al. [11] reported that high exposure to endotoxins was associated with reports of diarrhea. Moreover, the bioaerosols from WWTPs were able to penetrate the final stages of the Andersen apparatus (size: 0.65–1.1 µm), which indicates that bioaerosol is able to penetrate the human lung [3]. Furthermore, human opportunistic pathogens were always found in the bioaerosol generated from WWTPs [4,12]. Therefore, various studies have been conducted to evaluate the biological risk of the bioaerosol generated from WWTPs [3,4,12].

The properties of the bioaerosol affect its biological risk [10,13,14]; these include number of bioaerosols, size of bioaerosol, survival of bioaerosol during dispersal, and types of microorganisms present in bioaerosol. Smaller bioaerosols have a better ability to penetrate the lung and have a longer suspension time in the air, which results in a higher biological risk [14]. Larger numbers of bioaerosols generated and better survival of the bioaerosol during dispersal also increase the biological risk. These characteristics of bioaerosols can be affected by the water quality. High concentrations of organic surface active material tend to decrease the production of aerosols [15]. High salinity levels tend to limit bubble coalescence and thus increase aerosolization [16]. Moreover, the presence of salt content in the suspension medium leads to an increase in the aerodynamic size of the bioaerosol [17]. With the emergence of pharmaceuticals and personal care products in the aqueous environment [18], antibiotic resistance genes have been detected in the bioaerosol generated from WWTPs [19].

However, the impact of water quality of wastewater on the bioaerosol survival is not clearly understood. Our previous study showed that the airborne survival of *Escherichia coli* was related to the fluidity of the membrane, and the *E. coli* cultured in a lower temperature, which had a higher membrane fluidity, had a lower airborne survival compared to those cultured in a higher temperature [20]. This provides insight into the investigation of impact of water quality on airborne survival of bacteria. The water quality in wastewater varies among different WWTPs due to the difference in the sewage influents [21,22]. For example, some coastal cities (e.g., Hong Kong) use seawater for toilet flushing, which results in a higher salinity and pH of wastewater [23]. Therefore, it would be interesting to know if the change in membrane fluidity (fatty acid composition) caused by the variation in water quality of wastewater affects the airborne survival of bacteria after aerosolization.

This study aimed to investigate the impact of culture conditions on *E. coli* airborne survival. We hypothesized that an increase in membrane fluidity induced by the culture conditions would lead to a decrease in *E. coli* airborne survival and vice versa. *E. coli* was cultured in different conditions, including growth pH, temperature, salinity, and the presence of Dieldrin. Then, the fatty acid profile and the reduction in airborne survival of the *E. coli* were compared. The correlation between the membrane fluidity and the reduction in airborne survival was evaluated as well.

## 2. Materials and Methods

### 2.1. Bacteria Strains

*E. coli* was selected as a model bacterium due to its extensive use in bioaerosol studies and its common occurrence in bioaerosol generated from WWTPs [3,4,12,24]. *E. coli* BW25113 were purchased from Coli Genetic Stock Center (CGSC, Yale University, New Haven, CT, USA) [25].

### 2.2. Growth Conditions

The experimental procedures were similar to our previous study [20]. Briefly, fresh cultures of *E. coli* were grown at 30 °C at 150 rpm to the stationary phase. The stationary phase was determined by using growth curves. To simulate the wastewater quality in Hong Kong, a synthetic wastewater medium was prepared according to Wang et al. [26] with modification. A stock solution was prepared with the main components of glucose (19.57 g/L), sodium acetate (26.1 g/L), yeast extract (9.786 g/L), NH_4_Cl (18.37 g/L), K_2_HPO_4_ (1.92 g/L), and KH_2_PO_4_ (0.72 g/L). Then, the stock solution was mixed with tap water proportionally (1:4.4 in volume). Then, different substances were added to the medium according to the need (Table 1). Next, the bacterial cells were washed with phosphate buffered saline (PBS, pH 7.4) twice and then transferred to a six-jet Collison nebulizer (BGI Inc., Houston, Texas, TX, USA) for nebulization.

### 2.3. Experimental Setup on Airborne Survival

**Figure 1:**
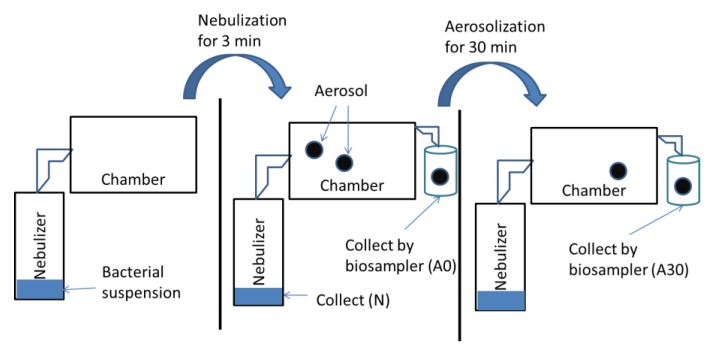
A schematic diagram of the setup and different sampling points. The bacterial suspension collected was called N. After completing the nebulization process, the aerosols were collected as A0, and the time was set as zero. Bioaerosols were suspended in the air for 30 min before sampling as A30. This schematic diagram was adopted from our previous study [20].

The reduction of bioaerosol survival of the bacteria was analyzed with procedures in our previous study with modification [20]. Each bacterial suspension was nebulized for 3 min in a nebulizer, and the aerosols generated were suspended in a cylindrical chamber (diameter × height: 50.8 cm × 58 cm, volume: 87 L) for 30 min. The air temperature and the relative humidity (RH) of 60% in the chamber were set based on Hong Kong climate conditions. The air temperature in the chamber was set at 30 ± 2 °C (average air temperature in a Hong Kong summer), and relative humidity was adjusted by either spraying sterile water or purging dehumidified air into the chamber to reach RH 60% (average lowest relative humidity in a Hong Kong summer). The temperature and the RH of the chamber were measured by a digital hygrometer (Lutron HT-3017, Taiwan). The experimental setup and the details are shown in a schematic diagram (Figure 1).

Bacteria in the nebulizer (N) and in the air (A0, A30) were sampled at different sampling points, as stated in our previous study [20]. Bacteria at A0 and A30 were collected by a biosampler (SKC, Inc., Eighty Four, PA, USA) at a flow rate of 12.5 L/min for 2 min. The biosampler was used to collect the bacteria, as the stress on bacteria induced by the biosampler was minimal due to less particle bounce [28,29]. The reduction in bacterial survival was calculated between different sampling points: (1) aerosolization (N to A0)—interface between bacteria in liquid suspension to the air; and (2) airborne suspension (A0 to A30)—before and after 30 min of airborne suspension. One sample was collected at each sampling point, and all the experiments were repeated in triplicate under well controlled laboratory conditions.

### 2.4. Determination of Bacterial Survival

To determine the bacterial survival, both culturable and DNA counts of the collected bacteria were measured. The plate-count method and the DNA counts by quantitative polymerase chain reaction (qPCR) were used for determination of the culturability of the bacterial cells [30]. In brief, bacterial DNA was extracted using a QIAamp DNA Mini Kit (Qiagen, Hilden, Germany) following the manufacturer’s protocol. The concentration of the extracted DNA samples was determined by qPCR using a QuantiNova^TM^ SYBR^®^ Green PCR Kit (Qiagen, Hilden, Germany) with the forward primer 784 (5′-GTGTGATATCTACCCGCTTCGC-3′) and the reverse primer 866 (5′-AGAACGGTTTGTGGTTAATCAGGA-3′), which bind to the *uidA* gene that is specific for *E. coli* determination [31].

To account for the potential physical loss of bioaerosols during aerosolization and suspension, survival was calculated as shown in Equation (1).

(1) $$\mathrm{The}\;\mathrm{survivial}=\;\frac{\mathrm{number}\;\mathrm{of}\;\mathrm{culturable}\;\mathrm{bacterial}\;\mathrm{cells}\;\left(\mathrm{plate}-\mathrm{count}\;\mathrm{data}\right)}{\mathrm{total}\;\mathrm{number}\;\mathrm{of}\;\mathrm{bacterial}\;\mathrm{cells}\;\left(\mathrm{qPCR}\;\mathrm{data}\right)}.$$

To assess the change in the airborne bacterial survival during aerosolization, the log reduction between the normalized culturable bacterial count before (N) and after 0 min (A0) of aerosolization was calculated as shown in Equation (2):

(2) $$Log\;reduction=\log_{10}N-\;\log_{10}A0.$$

Similar to aerosolization, to assess the change in the airborne bacterial survival during suspension, the log reduction between after 0 min (A0) and 30 min (A30) of aerosolization was calculated as shown in Equation (3):

(3) $$Log\;reduction=\log_{10}A0-\;\log_{10}A30.$$

It is possible there was no airborne bacterial survival after suspension; therefore, the actual value of log reduction could not be detected.

### 2.5. Fatty Acid Analysis

The fatty acid composition of the bacteria in culture medium was determined by MIDI Sherlock^®^ microbial identification system (MIDI, Inc., Newark, NJ, USA), as stated in our previous study [20]. The fatty acids were extracted and methylated according to the protocol recommended by the company (MIDI, Inc., Newark, NJ, USA). The extracted fatty acids were analyzed by an Agilent HP 6890 Series II gas chromatograph (Hewlett Packard, Avondale, AZ, USA) coupled with a flame ionization detector (FID). The fluidity index (FI) of the membrane was calculated according to Equation (4) [32]. All the results were the mean of duplicates.

(4) $$FI=\;\frac{UFAs+CFAs}{SFAs}$$

where UFAs are unsaturated fatty acids (16:1 and 18:1), CFAs are cyclopropane fatty acids (17:0 cyclo and 19:0 cyclo), and SFAs are saturated fatty acids (16:0 and 18:0)

### 2.6. Statistical Analysis

One-way analysis of variance (ANOVA) with Duncan’s post-hoc test (SPSS v. 23, IBM, Chicago, IL, USA) were used for determining and comparing the log reduction of the survival between different sampling points. Our data in this study proved not to violate the assumption of ANOVA, including normally distributed, independence of cases, and homogeneity of variance. The correlation between FI and log reduction of the survival was analyzed with Pearson correlation analysis (SPSS v. 23, IBM, Chicago, IL, USA). Differences between means with *p*-values lower than 0.05 (*p* < 0.05) were regarded as statistically significant. Otherwise, *p*-values lower than 0.05 (*p* < 0.05) were regarded as statistically correlated.

## 3. Results and Discussion

### 3.1. Impact of Culture Conditions on the Airborne Survival of E. coli

**Figure 2:**
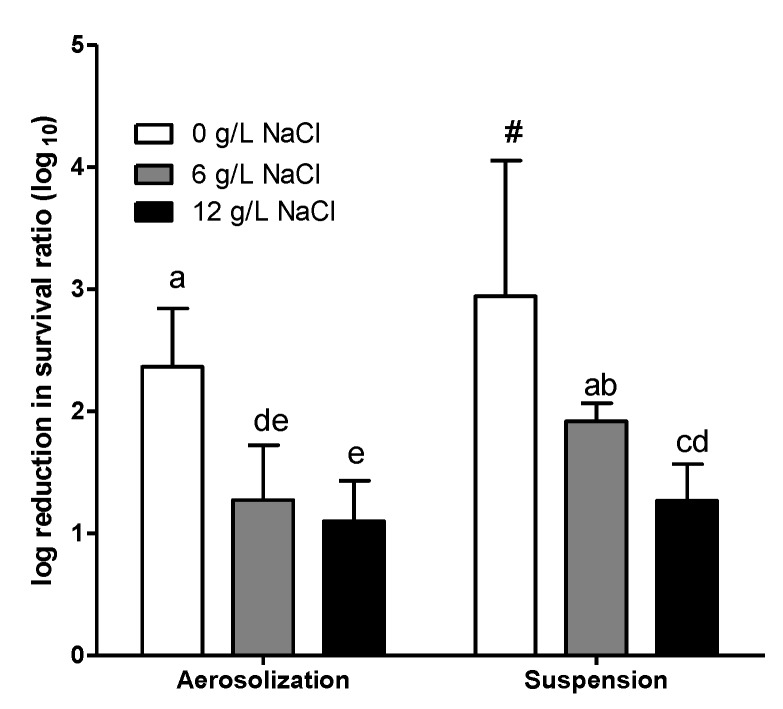
The log reduction in airborne survival of *E. coli* grown in synthetic wastewater medium with different salinity (addition of NaCl). Chamber temperature = 30 °C; chamber relative humidity (RH) = 60%. Error bars represent the standard deviation of replicates (*n* ≥ 3). The log reduction of different *E. coli* was statistically analyzed by one-way ANOVA. Grouping was conducted with post-hos test Duncan analysis, and the letters above the bars represent different grouping. The survival of *E. coli* cultured at 0 g/L NaCl collected at 30 min (A30) was zero. Therefore, the actual reduction may have been even higher than this.

Increase in NaCl concentration in the synthetic wastewater medium reduced the log reduction of survival for both aerosolization and airborne suspension (Figure 2). The log reduction of survival during aerosolization reduced from 2.37 (0 g/L) to 1.27 (6 g/L) and 1.10 (12 g/L). The effect was more remarkable for airborne suspension. The log reduction of survival of *E. coli* at 0 g/L growth salinity during airborne suspension was high (>2.94, and the acute value may have been even higher, as no airborne bacterial survival could be detected at A30). Then, the log reduction decreased significantly to 1.92 and 1.27 when the growth salinity increased to 6 and 12 g/L, respectively.

**Figure 3:**
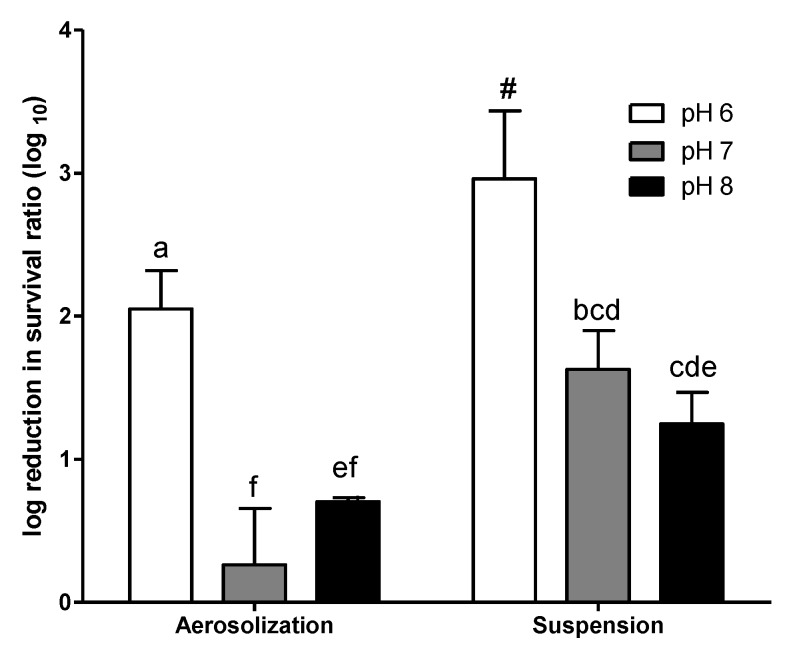
The log reduction in airborne survival of *E. coli* grown in synthetic wastewater medium with different pH (50 mM phosphate buffer). Chamber temperature = 30 °C; chamber RH = 60%. Error bars represent the standard deviation of replicates (*n* ≥ 3). The log reduction of different *E. coli* was statistically analyzed by one-way ANOVA. Grouping was conducted with post-hoc test Duncan analysis, and the letters above the bars represent different grouping. The survival of *E. coli* cultured at pH 6 collected at 30 min (A30) was zero. Therefore, the actual reduction may have been even higher than this.

The *E. coli* showed a higher reduction in survival when it grew at pH 6 (Figure 3). No significant difference in log survival was observed for *E. coli* grown at pH 7 and pH 8 for either aerosolization or airborne suspension. The log reduction of survival during aerosolization (2.05) and suspension (>2.97, and the acute value may have been even higher, as no airborne bacterial survival could be detected at A30) for *E. coli* grown at pH 6 was remarkably higher that those grown at pH 7 and pH 8.

**Figure 4:**
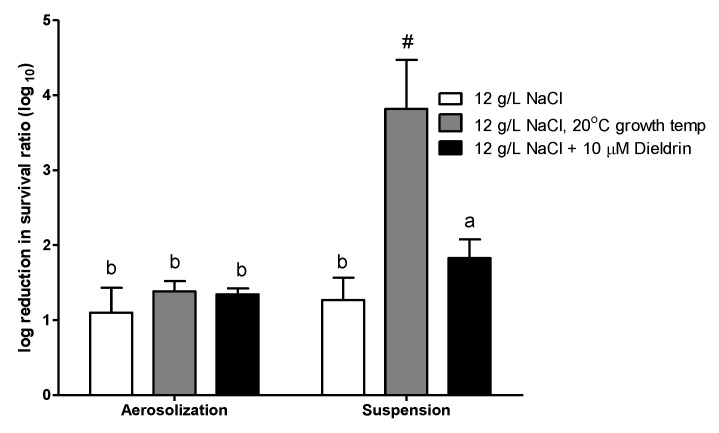
The log reduction in airborne survival of *E. coli* grown in synthetic wastewater medium with 12 g/L NaCl at 30 °C with the addition of 10 uM Dieldrin or cultured at 20 °C without Dieldrin. Chamber temperature = 30 °C; chamber RH = 60%. Error bars represent the standard deviation of replicates (*n* ≥ 3). The log reduction of different *E. coli* was statistically analyzed by one-way ANOVA. Grouping was conducted with post-hos test Duncan analysis, and the letters above the bars represent different grouping. The survival of *E. coli* cultured at 20 °C collected at 30 min (A30) was zero. Therefore, the actual reduction may have been even higher than this.

The effect of growth temperature and the addition of pesticide on airborne survival is shown in Figure 4. Addition of 10 μM Dieldrin in the synthetic wastewater medium did not affect the airborne survival of *E. coli* during aerosolization, but the log reduction of the survival was higher during suspension. The log reduction of the survival slightly increased to 1.83. For *E. coli* cultured at a lower temperature (20 °C), no significant effect was observed on the airborne survival of *E. coli* during aerosolization. However, the log reduction in survival during suspension was much higher (>3.81, and the acute value may have been even higher, as the survival at A30 was 0) than the *E. coli* grown at 30 °C.

### 3.2. Relation between Culture Conditions, Membrane Fluidity, and Airborne Survival

The percentage of different types of fatty acid profiles of *E. coli* cultured under different conditions is shown in Table 2. The details of each fatty acid can be found in Table S1 in Supporting Information. The culture conditions influenced the fatty acid profile differently. Increasing NaCl concentration in the synthetic wastewater medium led to a decrease in FI from 2.1 (0 g/L) to 1.45 (6 g/L) and 1.37 (12 g/L). The FIs of *E. coli* cultured in pH 7 (1.37) and pH 8 (1.38) were similar but increased remarkably when the pH decreased to 6 (1.74). The addition of 10 μM Dieldrin slightly decreased the FI (1.28). *E. coli* cultured at 20 °C resulted in an increase in FI (1.64). The changes in the fatty acid profile of *E. coli* cultured in different culture conditions agreed with the results of previous studies. For example, Casadei et al. [32] reported that an increase in growth temperature led to a decrease in *E. coli* membrane fluidity. The ratio of saturated fatty acid increased with increasing growth salinity, as stated by James and Armstrong [33]. Increase in growth pH resulted in an increase in unsaturated fatty acid and a decrease in cyclopropane fatty acid [34]. Rosas et al. [27] reported that the addition of 10 μM Dieldrin in the medium resulted in a higher membrane saturation of *E. coli*.

Our previous study reported a significant correlation between the FI of *E. coli* and the log reduction of survival during airborne suspension at 20 °C and RH 60% [20]. This correlation was also observed in this study in both aerosolization and suspension. A significant correlation was observed between the FI and the log reduction in survival during aerosolization and suspension (Table 3). The results agreed with our previous study and further confirmed the importance of membrane fluidity in regard to the airborne survival of *E. coli*. Reduction in FI resulted in a tightly packed membrane and could protect the bacteria to deal with the dehydration process during aerosolization and airborne suspension [20]. As the higher air temperature (30 °C) imposed a higher stress to the *E. coli* (bacteria airborne survival is usually higher with temperature >24 °C) [35], the protective effect of membrane fluidity became more significant, and thus the correlation was also observed for the aerosolization process.

Moreover, for *E. coli* cultured in conditions that had a FI > 1.6 (e.g., 20 °C, pH 6, and 0 g/L), the reduction in survival was significantly higher (all the *E. coli* died at A30 and survival dropped to zero). In our previous study, the *fabR* mutant (FI = 0.61) showed a high resistance (almost no reduction in survival) when the *E. coli* was suspended at air temperature 20 °C and RH 60% [20]. These results suggested that there was a critical fluidity (FI) value for the *E. coli* that led to hypersensitivity (for high FI) and hyper resistance (for low FI) of *E. coli* to airborne suspension.

Although bacterial membrane fluidity is a major factor in regard to airborne survival, the results suggested that other physiological changes of *E. coli* may also have affected the airborne survival. For example, *E. coli* cultured in the presence of Dieldrin had a lower FI but also had a slightly higher reduction in survival during suspension. *E. coli* cultured at pH 7 and pH 8 and 12 g/L NaCl had similar FIs but showed a difference in reduction of survival during aerosolization and suspension. Other factors such as oxidative stress protection may also have affected the survival, which requires more research.

### 3.3. Environmental Implication

Airborne survival of bacteria is determined by many factors. The impact of meteorological factors, especially RH and air temperature, is well known [35]. The effect of suspension medium on the airborne survival of bacteria has been reported as well [17]. This study provides a new aspect for assessment of bioaerosol survival in different wastewaters. A link between the culture conditions, the bacterial physiology, and the airborne survival was established. Besides meteorological factors, water quality of wastewater can also be used to evaluate the airborne survival of bacteria. Water quality of wastewater (culture conditions) that leads to low membrane fluidity of bacteria increases the airborne survival of bioaerosol and vice versa.

The impacts of culture conditions such as growth temperature, pH, and salinity on bacterial membrane fatty acid were well studied. For example, bacteria cultured in a low temperature and a high osmotic pressure (e.g., salinity) generally had high membrane fluidity [36]. Now, the information can be used to predict the airborne survival of the bacteria. For example, bacteria in bioaerosols generated from wastewater with a higher salinity (e.g., Hong Kong, ranging from 7–12 g/L Cl^−^) is very likely to have low membrane fluidity and thus a higher survival than those generated in a WWTP with a low salinity (e.g., <1 g/L Cl^−^). This concept can be incorporated into the current assessment based on the meteorological factors to improve the prediction of bioaerosol survival.

This study was conducted in laboratory conditions with a limited number of experiments, which limited the extrapolation of the results to the real situation. More factors that may have affected the FI of bacteria should be investigated. Moreover, bacterial strains and wastewater should be collected from real environments in a future study to further validate the findings of this study.

## 4. Conclusions

This study reveals the relationship between culture conditions, bacterial membrane fluidity, and bio-airborne survival. *E. coli* were cultured under different conditions, including growth pH, temperature, salinity, and the presence of Dieldrin, and then the fatty acid profile of the *E. coli* cultures was determined.

In brief, an increase in NaCl concentration in the synthetic wastewater medium reduced the log reduction of survival for both aerosolization and airborne suspension. On the other hand, the *E. coli* showed a higher reduction in survival when it grew at pH 6.

*E. coli* cultured at a lower temperature (20 °C) or with the addition of 10 μM Dieldrin in the synthetic wastewater medium showed no significant effects on the airborne survival of *E. coli* during aerosolization. However, the log reduction in survival was higher during suspension.

The fatty acid profile and the reduction in airborne survival of the *E. coli* were investigated and compared. The results showed that the culture conditions that led to low membrane fluidity resulted in a lower reduction in the airborne survival of *E. coli*. and vice versa. This provides new insight into the evaluation of bioaerosol survival and the assessment of the biological risks of bioaerosols.

## Figures and Tables

**Table 1:** Details of the tested culture conditions in this study.

Tested Conditions	Tested Range	Procedures	Justification
Salinity	0, 6, and 12 g/L NaCl	Addition of NaCl into the synthetic wastewater medium.	Salinity of wastewater was usually <10 g/L. Salinity of Hong Kong wastewater ranged from 7–12 g/L Cl^−^ due to the use of seawater for toilet flushing [23].
pH	pH 6, 7, and 8	Addition of appropriate amounts of K_2_HPO_4_ and KH_2_PO_4_ into the synthetic wastewater medium according to the phosphate buffer formulation to achieve desired pH (ionic strength 50 mM).	Normal wastewater ranged from 6–8. The pH of Hong Kong wastewater ranged from 7–8 [23].
Temperature	20 and 30 °C	Cultured the bacteria in synthetic wastewater medium with 12 g/L NaCl at different temperatures.	Culture the bacteria in synthetic wastewater medium with 12 g/L NaCl at different temperatures. The average air temperatures in Hong Kong are 20 and 30 °C
Presence of organic pollutants	10 μM Dieldrin	Addition of Dieldrin into the synthetic wastewater medium with 12 g/L NaCl.	Addition of 8.6 μM Dieldrin significantly decreased the fluidity of *E. coli* cell membrane [27].

**Table 2:** Percentage of unsaturated fatty acids (UFAs), saturated fatty acids (SFAs), cyclopropane fatty acid (CFA), and fluidity index (FI) of the *E. coli* cultured at different conditions.

	0 g/L NaCl	6 g/L NaCl	12 g/L NaCl	12 g/L NaCl + 10 uM Dieldrin	12 g/L NaCl (Grown at 20 °C)	pH 6	pH 7	pH 8
UFAs ^a^	37.0	16.2	14.0	13.9	30.9	40.7	19.6	32.1
SFAs ^b^	26.8	32.8	34.6	32.7	31.2	32.0	32.3	32.4
CFAs ^c^	19.2	31.4	33.4	28.0	20.5	15.0	24.8	12.6
FI ^d^	2.10	1.45	1.37	1.28	1.65	1.74	1.37	1.38

^a^ UFAs = 16:1 + 18:1; ^b^ SFAs = 16:0 + 18:0; ^c^ CFAs = 17:0 cyclo + 19:0 cyclo; ^d^ FI = (UFAs + CFAs)/SFAs, All values were mean of duplicates.

**Table 3:** Pearson correlation between membrane FI and bacterial survival during aerosolization and airborne suspension for *E. coli* cultured in different conditions. *p* < 0.05 is regarded as significantly correlated.

	Aerosolization	Suspension
Number of sample	24	24
Pearson correlation	0.8025	0.5606
*p* value (two-tailed)	*p* < 0.0001	0.0044

## Supplementary Materials

The following are available online at https://www.mdpi.com/1660-4601/16/23/4745/s1, Table S1: Fatty acid composition of the *E. coli* cultured at different conditions.

## References

[1] Hultin K.A.H., Krejci R., Pinhassi J., Gómez-Consarnau L., Mårtensson E.M., Hagström Å., Nilsson E.D., Gomez-Consarnau L., Mårtensson M., Nilsson D. (2011). Aerosol and bacterial emissions from Baltic Seawater. Atmos. Res..

[2] Sialve B., Gales A., Hamelin J., Wery N., Steyer J.-P. (2015). Bioaerosol emissions from open microalgal processes and their potential environmental impacts: What can be learned from natural and anthropogenic aquatic environments?. Curr. Opin. Biotechnol..

[3] Brandi G., Sisti M., Amagliani G. (2000). Evaluation of the environmental impact of microbial aerosols generated by wastewater treatment plants utilizing different aeration systems. J. Appl. Microbiol..

[4] Han Y., Li L., Liu J. (2013). Characterization of the airborne bacteria community at different distances from the rotating brushes in a wastewater treatment plant by 16S rRNA gene clone libraries. J. Environ. Sci..

[5] Douwes J., Thorne P., Pearce N., Heederik D. (2003). Bioaerosol Health Effects and Exposure Assessment: Progress and Prospects. Ann. Occup. Hyg..

[6] Walser S.M., Gerstner D.G., Brenner B., Bünger J., Eikmann T., Janssen B., Kolb S., Kolk A., Nowak D., Raulf M. (2015). Evaluation of exposure—Response relationships for health effects of microbial bioaerosols—A systematic review. Int. J. Hyg. Environ. Health.

[7] Farokhi A., Heederik D., Smit L.A.M. (2018). Respiratory health effects of exposure to low levels of airborne endotoxin—A systematic review. Environ. Health.

[8] Blair A., Zahm S.H., Pearce N.E., Heineman E.F., Fraumeni J.F. (1992). Clues to cancer etiology from studies of farmers. Scand. J. Work. Health.

[9] Khuder S.A., Mutgi A.B., Schaub E.A. (1998). Meta-analyses of brain cancer and farming. Am. J. Ind. Med..

[10] Kim K.-H., Kabir E., Jahan S.A. (2018). Airborne bioaerosols and their impact on human health. J. Environ. Sci..

[11] Ulla I.I., Niels O.B., Niels E., Birgitte H.N., Otto M.P., Helle W. (1999). Exposure-response Relationship between Gastrointestinal Problems among Waste Collectors and Bioaerosol Exposure. Scand. J. Work Health.

[12] Carducci A., Tozzi E., Rubulotta E., Casini B., Cantiani L., Rovini E., Muscillo M., Pacini R. (2000). Assessing airborne biological hazard from urban wastewater treatment. Water Res..

[13] Griffin D. (2007). Atmospheric Movement of Microorganisms in Clouds of Desert Dust and Implications for Human Health. Clin. Microbiol. Rev..

[14] Gralton J., Tovey E., McLaws M.-L., Rawlinson W.D. (2011). The role of particle size in aerosolised pathogen transmission: A review. J. Infect..

[15] Marks R., Kruczalak K., Jankowska K., Michalska M. (2001). Bacteria and fungi in air over the Gulf of Gdańsk and Baltic sea. J. Aerosol Sci..

[16] Mårtensson E.M., Nilsson E.D., De Leeuw G., Cohen L.H., Hansson H.C. (2003). Laboratory simulations and parameterization of the primary marine aerosol production. J. Geophys. Res. Atmos..

[17] Lai K.M., Burge H.A., First M.W. (2004). Size and UV Germicidal Irradiation Susceptibility of Serratia marcescens when Aerosolized from Different Suspending Media. Appl. Environ. Microbiol..

[18] Sui Q., Huang J., Deng S., Chen W., Yu G. (2011). Seasonal variation in the occurrence and removal of pharmaceuticals and personal care products in different biological wastewater treatment processes. Environ. Sci. Technol..

[19] Li J., Zhou L., Zhang X., Xu C., Dong L., Yao M. (2016). Bioaerosol emissions and detection of airborne antibiotic resistance genes from a wastewater treatment plant. Atmos. Environ..

[20] Ng T., Chan W., Lai K. (2018). Influence of membrane fatty acid composition and fluidity on airborne survival of *Escherichia coli*. Appl. Microbiol. Biotechnol..

[21] Singh K.P., Mohan D., Sinha S., Dalwani R. (2004). Impact assessment of treated/untreated wastewater toxicants discharged by sewage treatment plants on health, agricultural, and environmental quality in the wastewater disposal area. Chemosphere.

[22] Westgate P.J., Park C. (2010). Evaluation of proteins and organic nitrogen in wastewater treatment effluents. Environ. Sci. Technol..

[23] Lee K.Y., Ng T.W., Li G., An T., Kwan K.K., Chan K.M., Huang G., Yip H.Y., Wong P.K. (2015). Simultaneous nutrient removal, optimised CO2 mitigation and biofuel feedstock production by Chlorogonium sp. grown in secondary treated non-sterile saline sewage effluent. J. Hazard. Mater..

[24] Karra S., Katsivela E. (2007). Microorganisms in bioaerosol emissions from wastewater treatment plants during summer at a Mediterranean site. Water Res..

[25] Baba T., Ara T., Hasegawa M., Takai Y., Okumura Y., Baba M., Datsenko K.A., Tomita M., Wanner B.L., Mori H. (2006). Construction of *Escherichia coli* K-12 in-frame, single-gene knockout mutants: The Keio collection. Mol. Syst. Biol..

[26] Wang J., Lu H., Chen G.-H., Lau G.N., Tsang W.L., van Loosdrecht M.C.M. (2009). A novel sulfate reduction, autotrophic denitrification, nitrification integrated (SANI) process for saline wastewater treatment. Water Res..

[27] Rosas S.B., Secco M., Ghittoni N.E. (1980). Effects of pesticides on the fatty acid and phospholipid composition of *Escherichia coli*. Appl. Environ. Microbiol..

[28] Lin X., Willeke K., Ulevicius V., Grinshpun S.A. (1997). Effect of Sampling Time on the Collection Efficiency of All-Glass Impingers. Am. Ind. Hyg. Assoc. J..

[29] Macher J.M. (1997). Evaluation of bioaerosol sampler performance. Appl. Occup. Environ. Hyg..

[30] Ng T., Chan W., Lai K. (2017). Importance of stress-response genes to the survival of airborne *Escherichia coli* under different levels of relative humidity. AMB Express.

[31] Frahm E., Obst U. (2003). Application of the fluorogenic probe technique (TaqMan PCR) to the detection of Enterococcus spp. and *Escherichia coli* in water samples. J. Microbiol. Methods.

[32] Casadei M.A., Manas P., Niven G., Needs E., Mackey B.M. (2002). Role of Membrane Fluidity in Pressure Resistance of *Escherichia coli* NCTC 8164. Appl. Environ. Microbiol..

[33] McGarrity J.T., Armstrong J.B. (1975). The effect of salt on phospholipid fatty acid composition in *Escherichia coli* K-12. BBA Lipids Lipid Metab..

[34] Marshall D. (2004). Adaptation of *Escherichia coli* O157:H7 to pH alters membrane lipid composition, verotoxin secretion, and resistance to simulated gastric fluid acid. Appl. Environ. Microbiol..

[35] Tang J.W. (2009). The effect of environmental parameters on the survival of airborne infectious agents. J. R. Soc. Interface.

[36] Los D.A., Murata N. (2004). Membrane fluidity and its roles in the perception of environmental signals. BBA Biomembr..

