# Supplementary material for: Airborne Survival of Escherichia coli under Different Culture Conditions in Synthetic Wastewater
Source: Int J Environ Res Public Health. 2019 Nov 27;16(23):4745. doi: 10.3390/ijerph16234745 (PMC6926559; doi:10.3390/ijerph16234745)
Supplement: Supplementary file 1 [file ijerph-16-04745-s001.docx]

Article

Airborne Survival of *Escherichia coli* under Different Culture Conditions in Synthetic Wastewater

Wing Lam Chan, Wing Tung Chung and Tsz Wai Ng *

Department of Biology, Hong Kong Baptist University, Kowloon Tong, Hong Kong; [winglam1112@gmail.com](mailto:winglam1112@gmail.com) (W.L.C.); [14221845@life.hkbu.edu.hk](mailto:14221845@life.hkbu.edu.hk) (W.T.C.)

***** Correspondence: [13208187@life.hkbu.edu.hk](mailto:13208187@life.hkbu.edu.hk)

Received: 13 October 2019; Accepted: 25 November 2019; Published: date

**Table S1.** Fatty acid composition (percentage) of the *E. coli* cultured at different conditions.

| **Fatty Acid (s).** | **0 g/L NaCl** | **6 g/L NaCl** | **12 g/L NaCl** | **12 g/L NaCl + 10 uM Dieldrin** | **12 g/L NaCl (Grow at 20 °C)** | **pH 6** | **pH 7** | **pH 8** |
| --- | --- | --- | --- | --- | --- | --- | --- | --- |
| 12:0 | 3.4 | 3.8 | 3.8 | 4.3 | 2.2 | 2.7 | 5.6 | 5.5 |
| 12:0 2OH | 0 | 0 | 0 | 0 | 0 | 0 | 0.5 | 0.1 |
| 12:0 3OH | 0 | 0 | 0 | 0 | 0 | 0 | 1.0 | 0.8 |
| 14:0 | 5.3 | 6.7 | 6.3 | 6.5 | 6.2 | 4.9 | 6.2 | 7.0 |
| 14:0 3OH | 8.4 | 9.0 | 7.8 | 10.4 | 5.0 | 4.6 | 7.2 | 7.5 |
| 16:1 w7c | 6.4 | 2.3 | 1.7 | 2.1 | 5.4 | 10.1 | 12.1 | 21.2 |
| 16:0 | 26.8 | 32.8 | 34.6 | 31.7 | 31.2 | 32.0 | 32.3 | 32.2 |
| 17:0 cyclo | 12.0 | 19.1 | 19.6 | 17.6 | 12.7 | 11.2 | 20.7 | 11.8 |
| 17:0 ISO 3OH | 0 | 0 | 0 | 4.3 | 0 | 0 | 0 | 0 |
| 17:0 10 methyl | 0 | 0 | 0 | 0 | 0 | 0 | 2.8 | 1.9 |
| 18:1 w9c | 0 | 0 | 0 | 0 | 0 | 0 | 0.6 | 1.2 |
| 18:1 w7c | 30.6 | 13.9 | 12.3 | 11.8 | 25.5 | 30.6 | 6.9 | 9.7 |
| 18:0 | 0 | 0 | 0 | 1.0 | 0 | 0 | 0 | 0.2 |
| 19:0 cyclo w8c | 7.2 | 12.3 | 13.8 | 10.4 | 7.8 | 3.8 | 4.1 | 0.8 |
